# Thermotolerant glycosyl hydrolases-producing *Bacillus aerius* CMCPS1 and its saccharification efficiency on HCR-laccase (LccH)-pretreated corncob biomass

**DOI:** 10.1186/s13068-020-01764-2

**Published:** 2020-07-14

**Authors:** Meena Ganesan, Remitha Mathivani Vinayakamoorthy, Sugitha Thankappan, Iniyakumar Muniraj, Sivakumar Uthandi

**Affiliations:** 1 Biocatalysts Lab., Department of Agricultural Microbiology, Tamil Nadu Agricultural University, Coimbatore, 641003 India; 2 Department of Crop Management, Kumaraguru Institute of Agriculture, Sakthi Nagar, Erode, 638315 India

**Keywords:** Glycoside hydrolases, *B. aerius*, Hot springs, β-Glucosidase, Cellulases, HCR-LccH, Pretreatment, Saccharification

## Abstract

**Background:**

The current production of bioethanol based on lignocellulosic biomass (LCB) highly depends on thermostable enzymes and extremophiles owing to less risk of contamination. Thermophilic bacterial cellulases are preferred over fungi due to their higher growth rate, presence of complex multi-enzymes, stability, and enhanced bioconversion efficiency. Corncob, underutilized biomass, ensures energy conservation due to high lignocellulosic and more fermentable sugar content. In the present study, the thermophilic bacterium *Bacillus aerius* CMCPS1, isolated from the thermal springs of Manikaran, Himachal Pradesh, India, was characterized in terms of its activity, stability, and hydrolytic capacity. A two-step process comprising: (i) a combined strategy of hydrodynamic cavitation reaction (HCR)-coupled enzymatic (LccH at 6.5 U) pretreatment for delignification and (ii) subsequent hydrolysis of pre-treated (HCR-LccH) corncob biomass (CCB) using a thermostable cocktail of CMCPS1 was adopted to validate the efficiency of the process. Some of the parameters studied include lignin reduction, cellulose increase, and saccharification efficiency.

**Result:**

Among the five isolates obtained by in situ enrichment on various substrates, *B. aerius* CMCPS1, isolated from hot springs, exhibited the maximum hydrolytic activity of 4.11. The GH activity of the CMCPS1 strain under submerged fermentation revealed maximum filter paper activity (FPA) and endoglucanase activity of 4.36 IU mL^−1^ and 2.98 IU mL^−1^, respectively, at 44 h. Similarly, the isolate produced exoglucanase and β-glucosidase with an activity of 1.76 IU mL^−1^ and 1.23 IU mL^−1^ at 48 h, respectively. More specifically, the enzyme endo-1,4-β-d glucanase E.C.3.2.1.4 (CMCase) produced by *B. aerius* CMCPS1 displayed wider stability to pH (3–9) and temperature (30–90 °C) than most fungal cellulases. Similarly, the activity of CMCase increased in the presence of organic solvents (118% at 30% acetone v/v). The partially purified CMCase from the culture supernatant of CMCPS1 registered 64% yield with twofold purification. The zymogram and SDS-PAGE analyses further confirmed the CMCase activity with an apparent molecular mass of 70 kDa. The presence of genes specific to cellulases, such as cellulose-binding domain *Cel*B, confirmed the presence of GH family 46 and β-glucosidase activity (GH3). The multifunctional cellulases of CMCPS1 were evaluated for their saccharification efficiency on laccase (LccH, a fungal laccase from *Hexagonia hirta* MSF2)-pretreated corncob in a HCR. The lignin and hemicelluloses removal efficiency of HCR-LccH was 54.1 and 6.57%, respectively, with an increase in cellulose fraction (42.25%). The saccharification efficiency of 55% was achieved with CMCPS1 multifunctional cellulases at 50 °C and pH 5.0.

**Conclusion:**

The multifunctional cellulase complex of *B. aerius* CMCPS1 is a potential biocatalyst for application in lignocellulosic biomass-based biorefineries. The saccharification ability of HCR-LccH-pretreated corncob at elevated temperatures would be an advantage for biofuel production from lignocellulosic biomass.

## Background

Lignocellulosic biomass (LCB)-based bioethanol pilot plants has been increasing worldwide due to concerns over climate change, energy conservation, and food security [1]. Of the several processes to produce bioethanol, the cost for saccharification of LCB is still extremely high and requires cellulases. The presently available industrial cellulases contain low levels of β-glucosidases to hydrolyze the cellobiose to glucose. In addition, lignocelluloses-based biorefineries prefer multi-beneficial cellulolytic enzymes with temperature tolerance, and broader stability to pH, metals, and solvents. Such multi-beneficial cellulolytic enzymes offer several advantages, such as tolerance to elevated temperatures as well as harsh operational processes, and hence reduce microbial contamination during saccharification [2]. Moreover, the increased reaction rate of enzymes reduces the viscosity of medium with a simultaneous increase in the diffusion of simple sugars from complex polysaccharides. Therefore, it is imperative to screen multi-beneficial cellulases to suit the industrial requirements [3, 4].

Multi-beneficial cellulases isolated from bacteria are preferred over fungal enzymes owing to their rapid growth rate, more extensive genetic diversity, and easy manipulation of their genetic makeup [5]. The majority of the bacterial species can produce endoglucanases to hydrolyze amorphous celluloses such as carboxymethyl cellulose (CMC). However, several of them cannot hydrolyze crystalline cellulose effectively [6]. A few *Bacillus* spp. possess endoglucanases with microcrystalline cellulose (Avicel)-degrading activity [7]. Furthermore, thermotolerant cellulases with β-glucosidase activity can overcome the rate-limiting steps in the saccharification process and thus increase the glucose yield [2, 8]. However, successful hydrolysis of biomass and synergistic action of cellulase depend on an optimum pretreatment process. Of the several pretreatment strategies, the biological method is promising, as there is no inhibitor formation, it requires less energy, and is eco-friendly. Several studies have suggested combined pretreatment methods due to their better delignification efficiency [9]. More recently, hydrodynamic cavitation technology coupled with an oxidative enzyme (laccase 6.5 U of *Trametes versicolor*) was one of the successfully employed LCB pretreatment [10]. Highly reactive radicals (H– and OH–) generated due to the cavitational effect degrade lignin moieties, although not optimal for targeting specific end products [11]. In HCR-laccase coupled process, both the biocatalyst and biomass are continuously circulated throughout the reactor. Laccase releases phenoxy radicals to remove recalcitrant fractions of lignocellulosic biomass and enhances delignification efficiency. The present study deals with multi-copper oxidase (LccH) from a hyper laccase-producing fungus *Hexagonia hirta* MSF2 [12] in a hydrodynamic cavitation reactor (HCR). Hence, the present study isolated multifunctional cellulases encompassing microcrystalline cellulose degradation, pH, temperature, and metal tolerance for enhanced saccharification of HCR-LccH-pretreated corncob biomass (CCB).

## Results

### Thermophilic glycosyl hydrolases-producing bacteria

To develop a robust biomass conversion process that works at slightly elevated temperatures, a biotrap-based in situ enrichment was performed to isolate thermophilic bacteria. Five bacterial isolates that grow at relatively high temperature (> 50 °C) were isolated from thermal springs (Manikaran [~ 95 °C]) of Himachal Pradesh, India, through an in situ enrichment of several lignocellulosic substrates, namely paddy straw, banana fiber, banana pseudostem, rice husk, and palm fronds. Among the five isolates screened for their cellulolytic potential, CMCPS1 showed the maximum hydrolytic capacity of 4.11 under the plate assay (Table 1), and was used for further studies.

**Table 1:** The hydrolytic capacity of the potential bacterial isolates for cellulolytic activity

Isolates	Hydrolytic capacity
CMCBF1	2.72 (± 0.46)^d^
CMCFA1	2.43 (± 0.47)^c^
CMCFB1	1.26 (± 0.54)^c^
CMCPS1	4.11 (± 0.31)^a^
CMCR1	3.27 (± 0.17)^b^

Values represent mean (± standard error) (*n* = 3) and the values representing the same alphabets are not significant from each other as determined by DMRT at *p* = 0.05

**Fig. 1:**
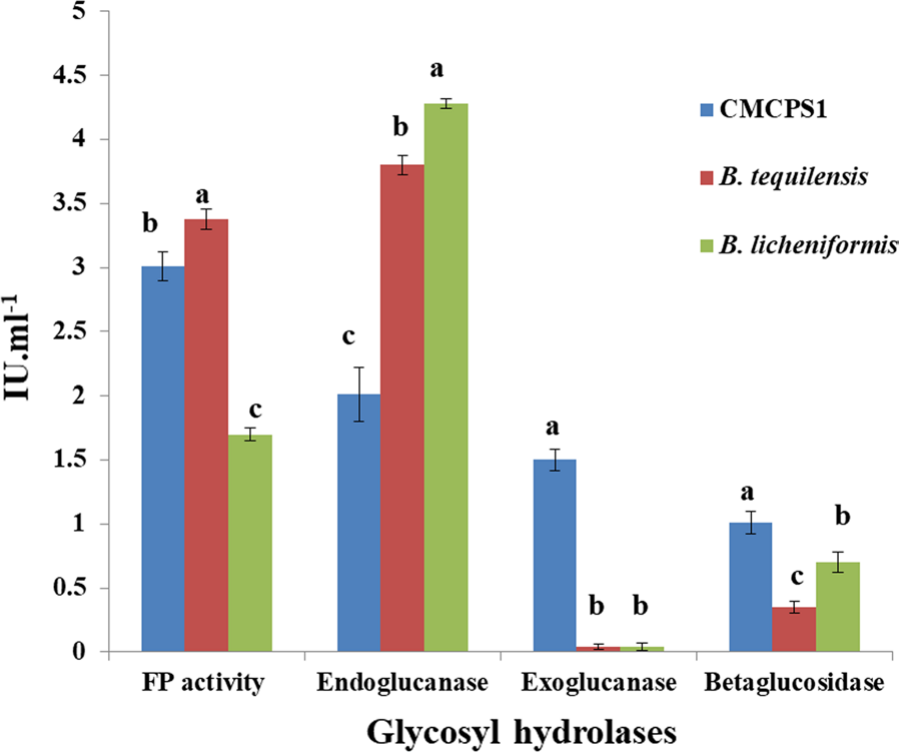
Cellulase production by thermophilic isolate CMCPS1 under submerged fermentation. Graph showing the cellulase (IU mL^−1^) activity of the isolate CMCPS1 in different assays, namely, filter paper (total cellulase), endoglucanase, exoglucanase, and β-glucosidase and compared with the standard cultures. The values represent mean of three replicates and significance tested by one-way ANOVA. The error bar signifies SE, and the mean values followed different lower case letters are significantly different from each other as determined by DMRT (*p *≤ 0.05)

Under submerged fermentation conditions, the potential isolate CMCPS1 showed a filter paper activity of 3.0 IU mL^−1^, endoglucanase activity of 2.0 IU mL^−1^, exoglucanase activity of 1.5 IU mL^−1^, and β-glucosidase activity of 1.0 IU mL^−1^ at 48 h (Fig. 1), which confirmed the significant production of extracellular glycoside hydrolases (GHs) using respective substrates. The exoglucanase and β-glucosidase activities of CMCPS1 were higher than those of *B. tequilensis* VCB1 and *B. licheniformis* KBFB3.

### Phylogenetic analysis of CMCPS1

**Fig. 2:**
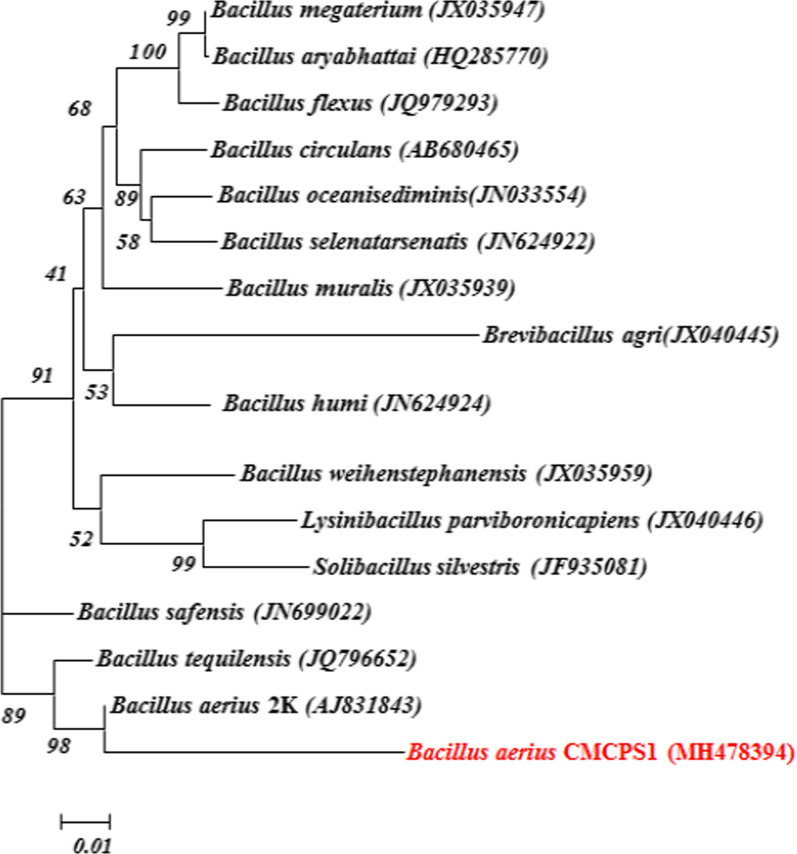
Phylogenetic tree for identification of the isolate CMCPS1. All sequences of the associated members of the genus *Bacillus* sp. were aligned with the sequences of CMCPS1. The tree was constructed using the 16S rRNA sequence retrieved from the database by using the neighbor-joining method. The bootstrap values were generated from 1000 replicates

The 16S rRNA gene sequence of the isolate CMCPS1 was subjected to a bioinformatic search for their nearest matching nucleotide sequence using the NCBI-BLAST analysis tool along with the type strains retrieved from NCBI databases. The phylogeny was generated using the neighborhood joining method with MEGA 7.0 and a bootstrap value of 0.02. The results revealed that the isolate was closely related to *B. aerius* and formed a clade with *B. aerius* 2K4, showing 98% sequence similarity. The sequence of the strain was deposited in the NCBI GenBank with an accession number of MH478394 (Fig. 2).

### Cellulase-specific genes and binding domains from *B. aerius* confirming GH activity

**Fig. 3:**
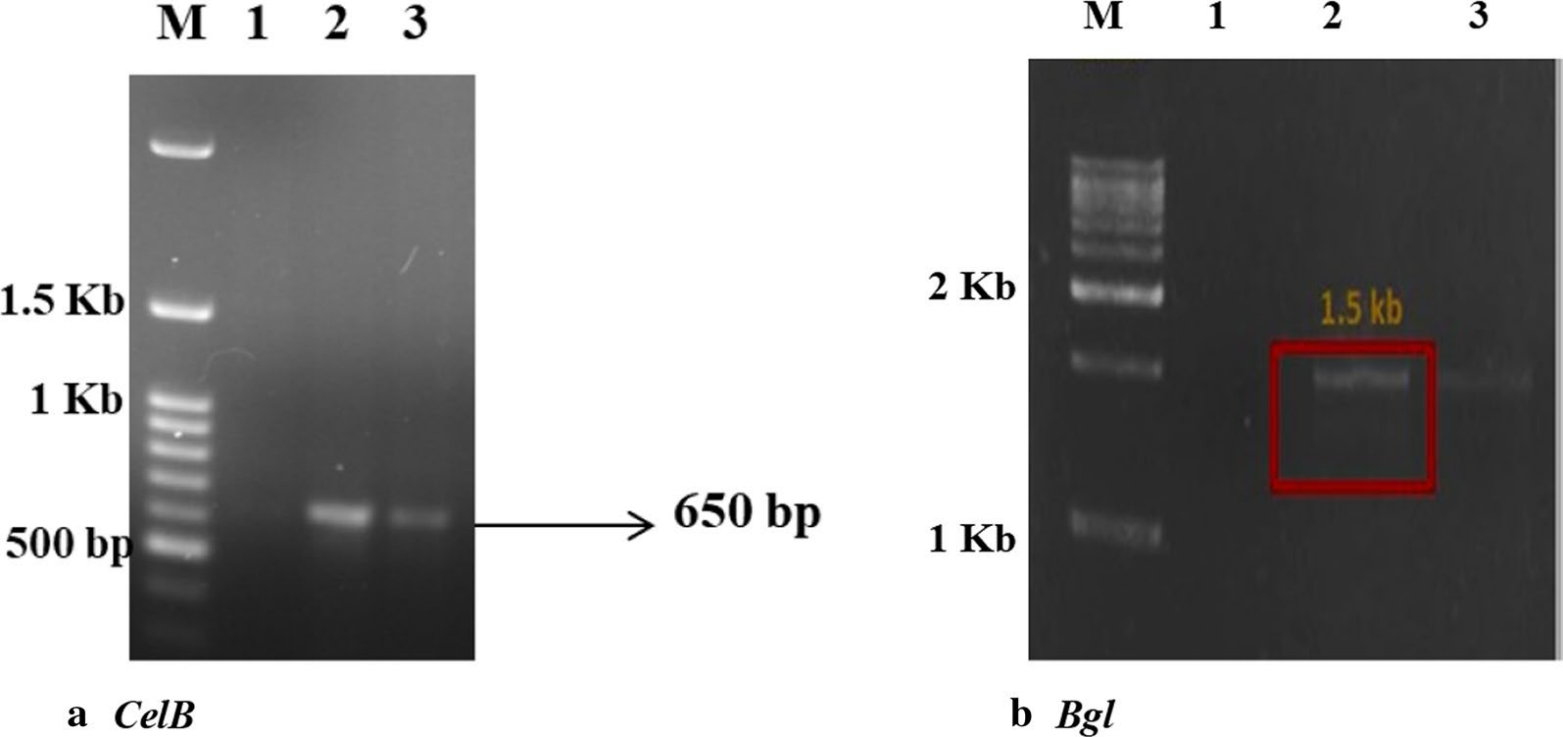
PCR-based confirmation of cellulase genes from *B. aerius* CMCPS1. **a***CelB* shows amplification at 650 bp, confirming the presence of cellulase producing region in the DNA. M—100 bp ladder, 1—negative control, 2—CMCPS1, 3—positive control (*B. licheniformis* KBFB3); **b***BGL* represents the presence of β-glucosidase in the size of 1.5 kb, M—1-kb molecular weight marker, 1—negative control, 2—CMCPS1, 3—positive control (*B. licheniformis* KBFB3)

The strain *B. aerius* CMCPS1 was screened using polymerase chain reaction (PCR) for cellulase GH46 (*celS* and *celB)* and β-glucosidase (GH3)-encoding genes (*bgl*). The results demonstrated that the strain harbored *CelB* corresponding to GH46, producing a 650-bp amplicon (Fig. 3a) in comparison to *B. licheniformis* KBFB2, used as a positive control. There was no amplification for the *celS* domain. Similarly, in β-glucosidase screening, a 1500-bp amplicon was observed (Fig. 3b), suggesting that the strain confirmed the presence of glycoside hydrolase activity compared with the standard culture *B. licheniformis* KBFB3 (NAIMCC-B-02118).

### Multi-GH complex of *B. aerius* CMCPS1

**Fig. 4:**
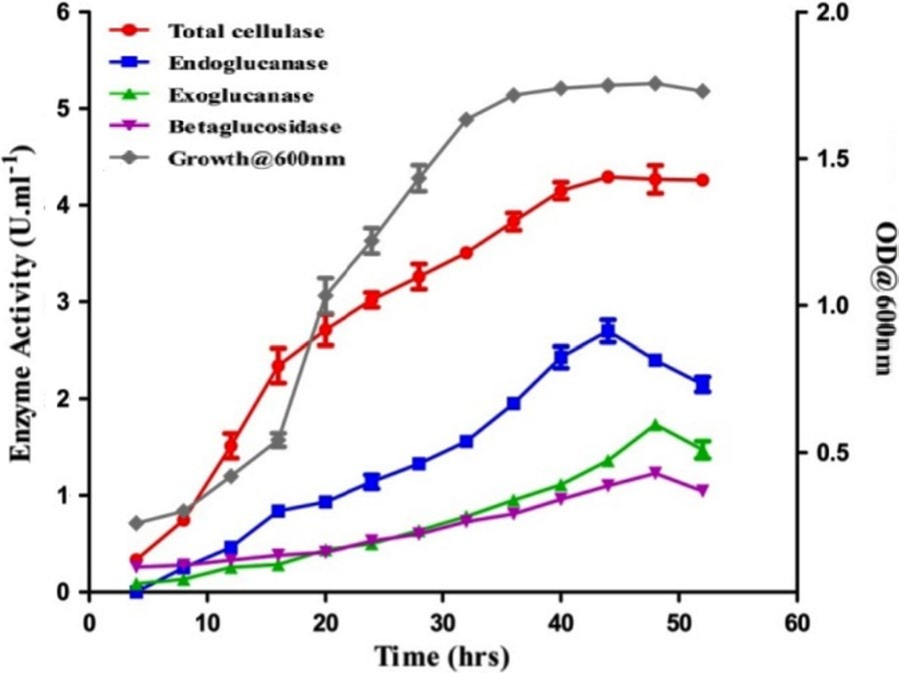
Time course cellulase production by *B. aerius* CMCPS1 on different substrates. Cellulase activity profile of CMCPS1 in different substrates viz., filter paper, Avicel, CMC, and cellobiose. The strain was grown in the production medium, incubated at 50 °C under submerged fermentation. The samples withdrawn at hourly intervals were subjected to enzyme assays using a standard protocol

Results of the FP activity assay of *B. aerius* CMCPS1 exhibited an activity of 4.36 IU mL^−1^ (Fig. 4). It was noted that as the growth (OD_600_) increased, FP units showed a pronounced and steady-state increase until the early stationary phase (44 h) and gradually declined thereafter toward the late stationary phase. The strain CMCPS1 showed carboxymethyl cellulase (CMCase E.C.3.2.1.4) activity of 2.98 IU mL^−1^at 48 h, which declined gradually (Fig. 4). The CMCase production and growth pattern are similar to that of FPA. The exoglucanase activity determined using Avicel (crystalline cellulose) as a substrate recorded a maximum titer of 1.76 IU mL^−1^ at 48 h of growth by CMCPS1. However, a drop in the activity was noticed after 48 h (Fig. 4). It was further noticed that unlike FPA and CMCase, the exoglucanase activity was maximum in the late stationary phase.

Cellobiase (E.C.3.2.1.21) activity or aryl β-glucosidase of the strain *B. aerius* CMCPS1 grown in a production medium containing cellobiose (1%) was assayed. Interestingly, the strain CMCPS1 showed an increasing trend up to 24 h, and attained a maximum titer of 1.23 IU mL^−1^ at 48 h; the activity declined thereafter (Fig. 4). Hence, β-glucosidase was constitutively produced during the logarithmic phase, and its production lasted until the late stationary phase.

### Characterization of CMCPS1 cellulase complex

**Fig. 5:**
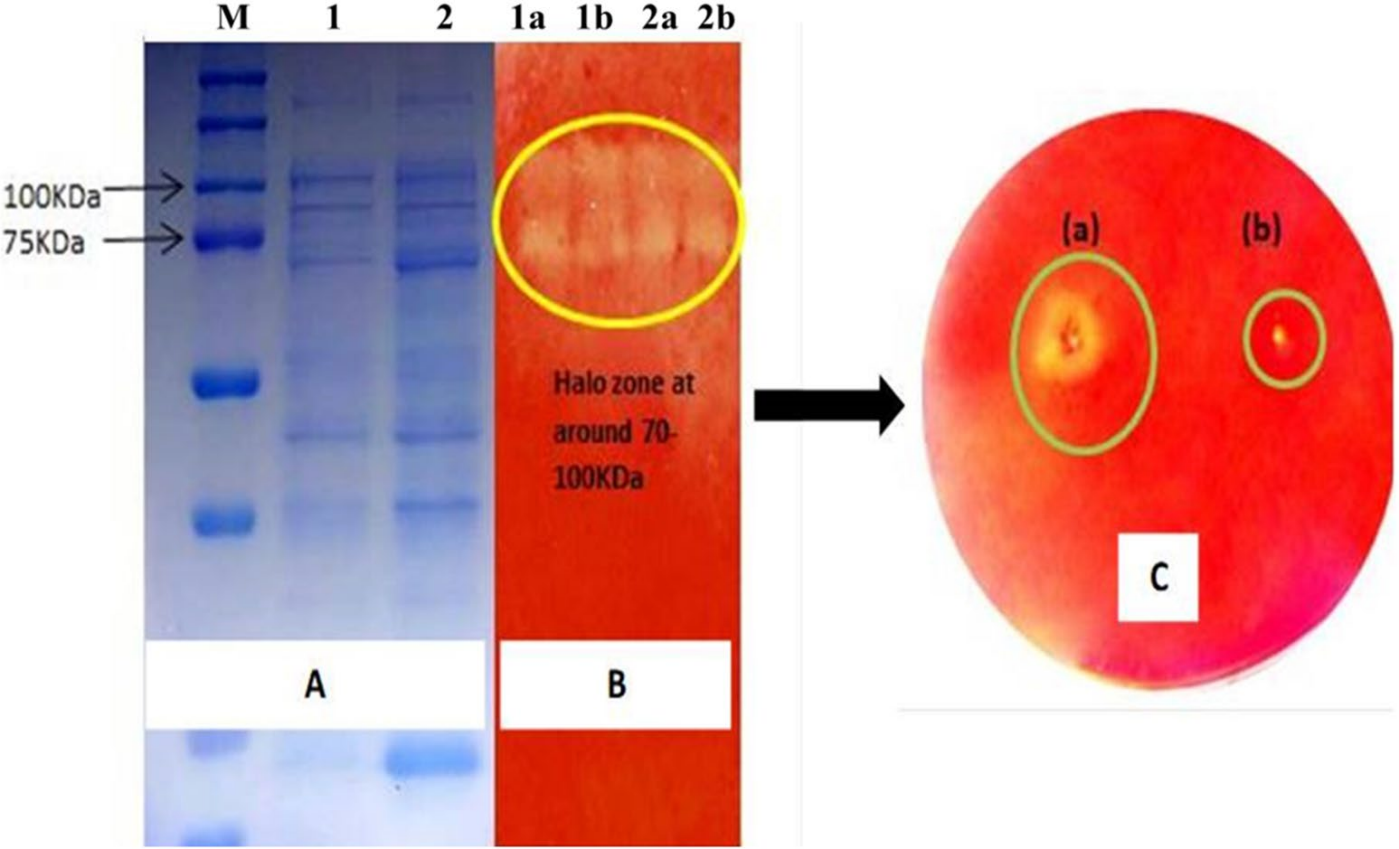
Protein profile of cellulase produced by *B. aerius* CMCPS1. SDS-PAGE and zymogram analysis showing cellulolytic activity. **A** SDS profile of cellulase produced by CMCPS1 grown on culture medium supplemented with CMC; M—marker; 1—positive check *B. licheniformis* KBFB3﻿; 2—CMCPS1. **B** In vitro zymogram of the cellulase activity of the purified enzyme; 1a and 1b—positive check *B. licheniformis* KBFB3 in two replicates; 2a and 1b—CMCPS1 in two replicates. **C** Gel diffusion assay of the partially purified cellulase (a) purified cellulase (b) sterile water control. The halo region indicates the cellulase activity of the *B. aerius* CMCPS1

Partially purified extracellular protein fraction of *B. aerius* CMCPS1 was subjected to SDS-PAGE analyses, and the results demonstrated three distinct bands of 100, 80, and 70 kDa (Fig. 5A). Native PAGE gel stained with Congo red revealed a clearing zone due to hydrolysis (Fig. 5B). Similarly, agarose well diffusion assay confirmed the CMCase activity (Fig. 5C).

### Optimal assay conditions and stability of CMCase

**Fig. 6:**
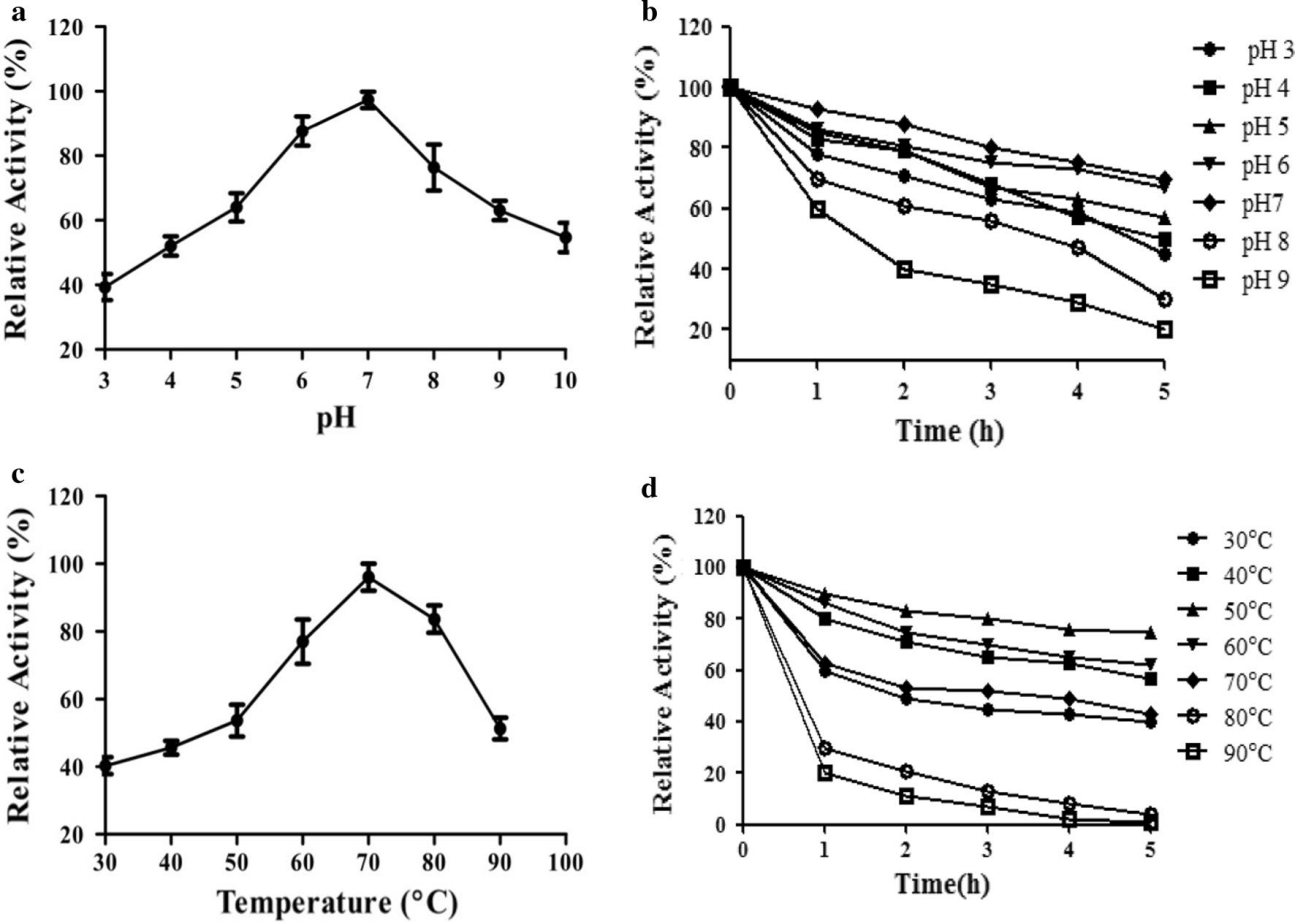
Optimal activity and stability of CMCase from *B. aerius* CMCPS1. **a** Effect of pH on enzyme activity, **b** effect of pH on enzyme stability, **c** effect of temperature on enzyme activity, and **d** effect of temperature on enzyme stability

The determination of biochemical characteristics of cellulase is of prime importance to elucidate the process parameters in industrial applications. The pH has a substantial role in cellulase activity; the maximum cellulase activity was observed at pH 7.0 in a phosphate buffer. Interestingly, partially purified cellulases retained more than 50% of their activity between pH 3.0 and 9.0 (Fig. 6a, b) after 5 h of incubation. Thermal inactivation of the enzyme is often encountered as a significant problem in most industrial processes. Industries prefer enzymes with thermophilic nature, as most of the operations in the industry work at moderately higher temperatures. The CMCase activity was maximum at 50 °C; thereafter, the activity reduced. The thermostability of CMCase from CMCPS1 evaluated by pre-incubation of the enzyme extract at pH 7.0 for 1 h demonstrated its moderate thermostability between 45 and 70 °C by retaining more than 80% of the activity. However, prolonged incubation of enzyme for 5 h drastically reduced the enzyme activity, retaining 50% of CMCase activity.

**Fig. 7:**
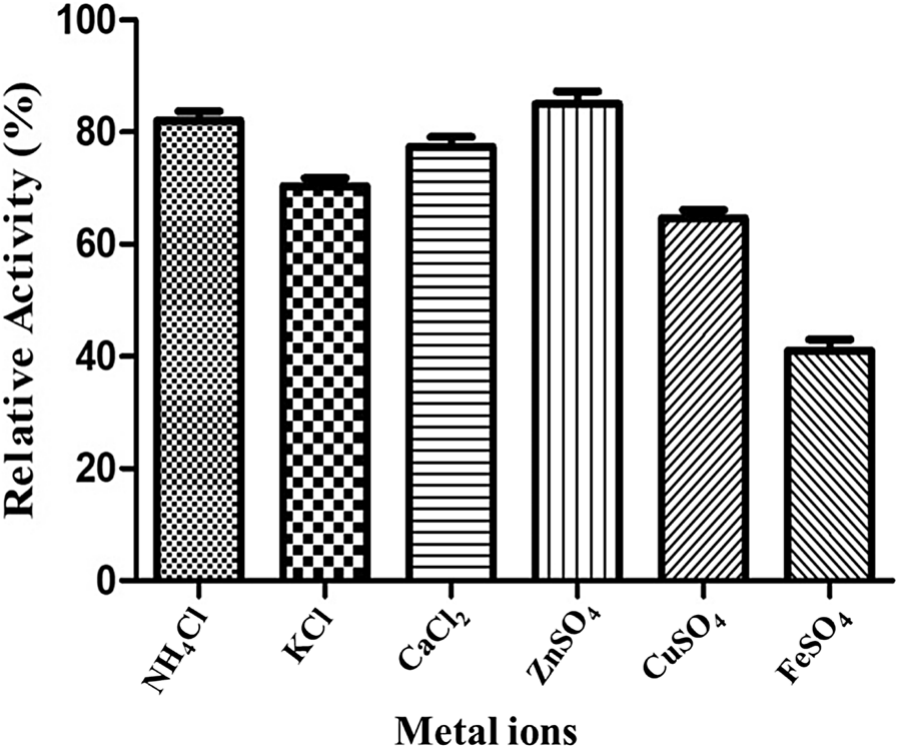
Effect of metal ions on CMCase activity. The activity was determined at 50 °C in the presence of metal ions (5 mM) and incubated for 1 h and assayed under standard conditions

**Fig. 8:**
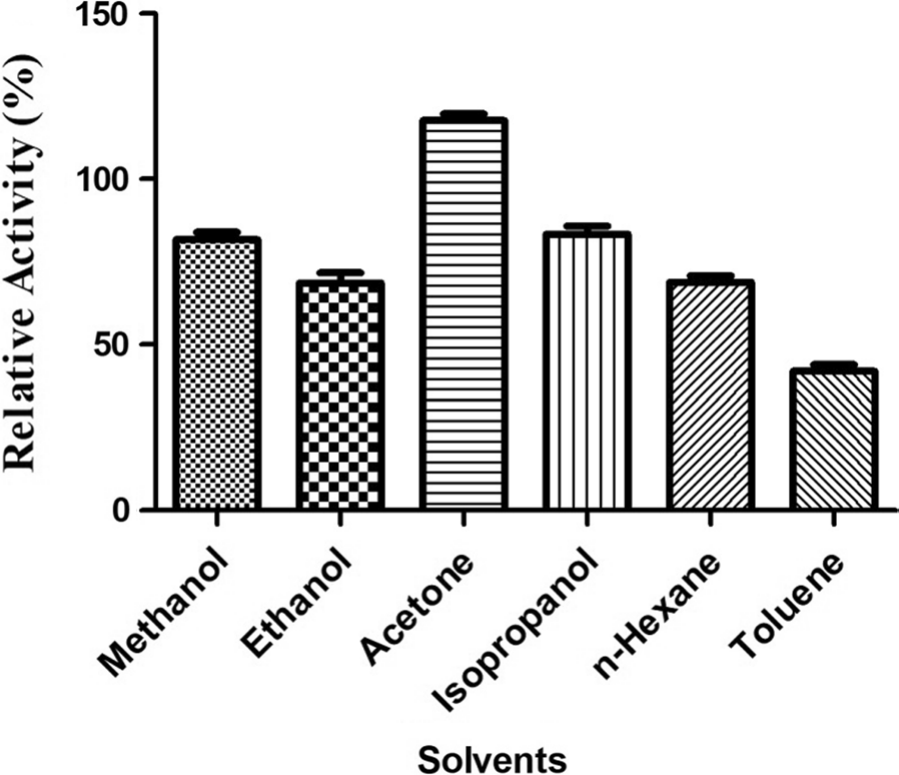
Effect of organic solvents on CMCase activity. The activity was determined at 50 °C in the presence of organic solvents (30% v/v) and incubated for 1 h and assayed under standard conditions

The CMCase activity on several metals showed that none of the metals activated the cellulase activity, whereas a distinct inhibition was noticed. Among the metal ions tested, the relative cellulase activity was more with Zn^2+^ (85%), followed by NH^4+^ (82%). Similarly, the results suggested that cellulase showed the minimum relative activity and was strongly inhibited in the presence of iron (65%) and copper. Further, potassium and calcium ions exhibited moderate inhibition of cellulase activity (70 and 77%, respectively). The effect of organic solvents (30% v/v) on the relative activity of cellulase showed that the enzyme is extraordinarily stable in the presence of acetone (117.73%) and moderately stable in methanol (81.66%) and isopropanol (83.33%). It was observed that ethanol and polar solvents, such as *n*-hexane and toluene, reduced the cellulase activity, as evidenced by the lower residual activities of 68.6, 68.7, and 41.96%, respectively (Figs. 7 and 8).

### Pretreatment of corncob biomass using HCR coupled with LccH

In the present study, CCB was pretreated with laccase (LccH at 6.5 U) from *H. hirta* MSF2 in an HCR for 60 min. The pretreatment helps to delignify the corncob biomass (CCB), depolymerize hemicellulose, and reduce cellulose crystallinity. Such a process will increase the porosity of the biomass, making CCB more amenable for the subsequent saccharification process. Here, the HCR-LccH pretreatment efficiently reduced the hemicellulose and lignin (6.57 and 54.1%, respectively), accompanied by an increase in cellulose (24.26%). The proximate fractions of both raw and HCR-LccH are depicted in Table 2.

**Table 2:** Chemical composition of raw and HCR-LccH-treated CCB

Composition	Raw CCB (%)	HCR-LccH treated CCB (%)	Efficiency (%)
Cellulose	34.00	42.25	24.26
Hemicellulose	27.38	25.58	6.57
Lignin	17.6	8.14	54.1
Extractives	18.62	14.78	–
Moisture	6.9	7.25	–
Ash	0.005	0.003	–

**Fig. 9:**
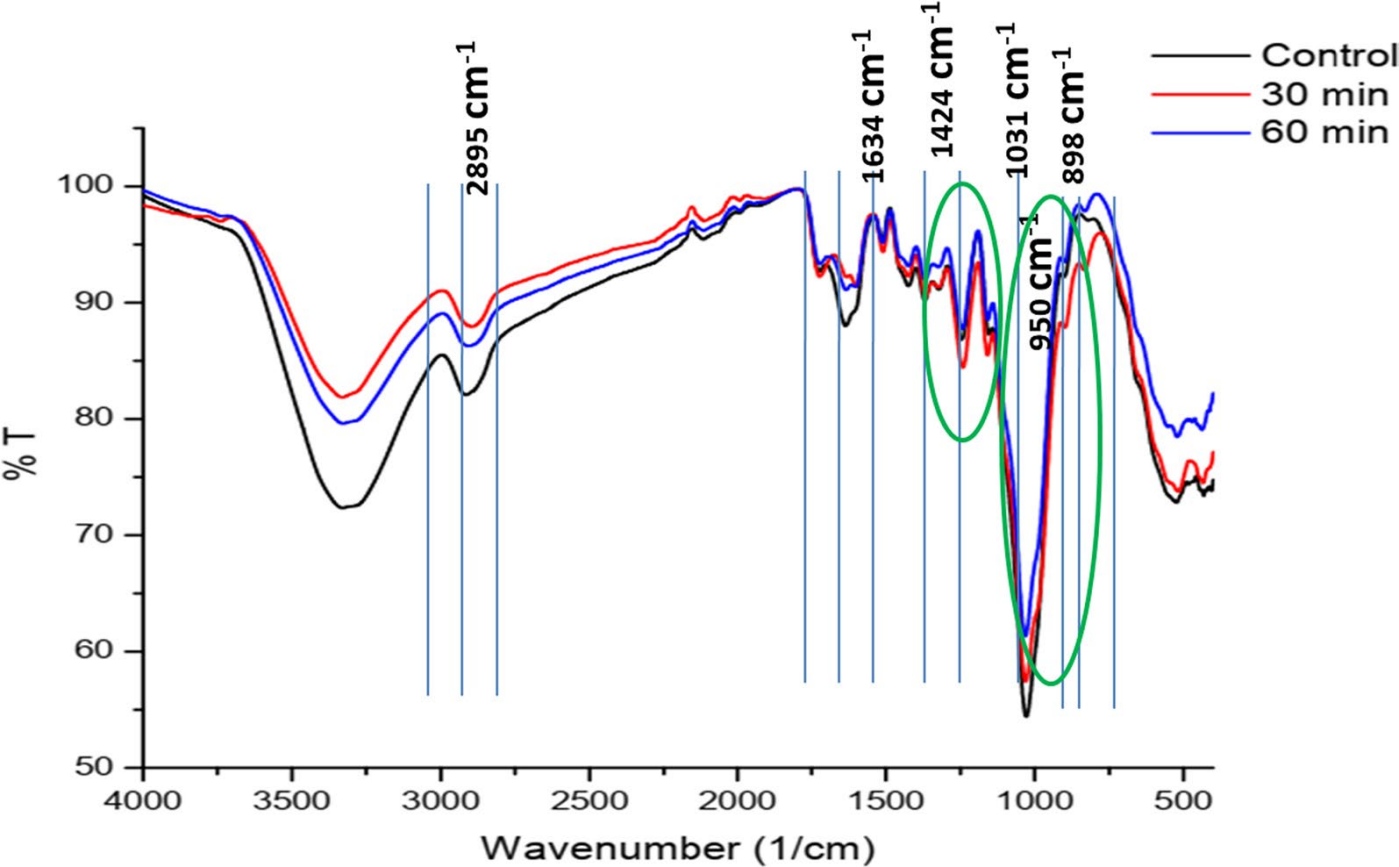
FT-IR spectrum of raw and HCR-LccH-pretreated corncob biomass at different time intervals. An increase in transmittance and the corresponding decrease in the depth of the spectrum refers a few bonds to absorb that particular wavelength. Cellulose increase represented by the wavelengths 950 (C–O), 1031 (C–O, C=C, C–C–O stretching) and 1424 (–CH_2_ vibration) cm^−1^, respectively. Strong vibration at 2895 cm^−1^ showed lignin reduction

The FT-IR spectral analysis of HCR-LccH-pretreated corncob samples revealed different wave numbers, functional groups, and their corresponding polymers that represented the presence of lignin, hemicelluloses, and cellulose structures. The absorption band peaks of cellulose, hemicelluloses, and lignin were shown to be stronger in the HCR-LccH-pretreated corncob samples compared to the untreated one. The performance of HCR-LccH pretreatment indicated significant changes in the structural polymers as compared to raw corncob. However, the pretreatment duration significantly influenced the lignin removal. A reduction in the peak intensity was observed at wavenumbers 950, 1031,1371, 1424, and 1634 cm^−1^ (Fig. 9). The peak at 898 cm^−1^ corresponds to the cleavage of β-glycosidic bonds in cellulose, whereas the bands at 1031 and 1100 cm^−1^ represent C–O–C vibrations associated with the pyranose ring and C–O–C asymmetric stretching in cellulose, respectively. Absorption spectra at 1371 and 1424 cm^−1^ represent C–H bending and symmetric CH_2_ bending, respectively, associated with cellulose scissoring. In addition, absorption peaks at 2895 and 3312 cm^−1^ indicate C–H stretching and –OH stretching, respectively, of intramolecular hydrogens within lignin and cellulose. The C=O stretching vibration and C–C vibration of the aromatic ring representing lignin were indicated by the absorption peaks at 1243 and 1514 cm^−1^, respectively. The acetyl groups in hemicelluloses and ester groups in lignin were represented by C=O vibrations in the band position of 1747 cm^−1^.

### Saccharification efficiency of cellulase from CMCPS1

**Fig. 10:**
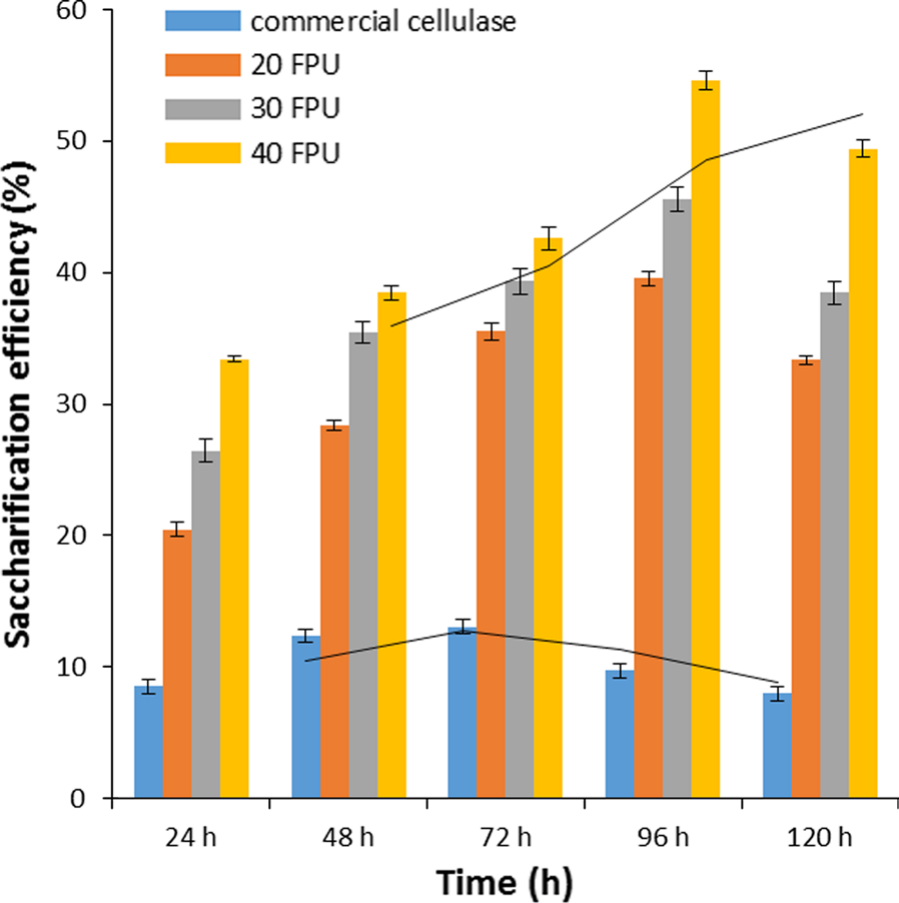
Saccharification efficiency of HCR-LccH-treated CCB using cellulase from *B. aerius* CMCPS1 compared with commercial cellulase of *Aspergillus niger*. Hydrolysis was performed at 50 °C for 120 h at 120 rpm, and the reducing sugars were estimated using dinitro salicylic acid reagent under standard assay conditions

After pretreatment, enzymatic saccharification was performed using cellulase from *B. aerius* CMCPS1, and the reducing sugar was estimated. The highest saccharification efficiency of 55% (reducing sugars of 1.37 g g^−1^) was obtained with 40 U cellulase per gram of dry biomass at a 5% solid loading rate. A gradual increase in the saccharification efficiency was observed, as the hydrolysis progressed up to 96 h, and after that, a sudden decline at 120 h was observed. At lower enzyme loading of 20 U g^−1^, 40% efficiency (0.99 g g^−1^ reducing sugars) was achieved in 96 h, however, doubling the enzyme load to 40 U g^−1^ increased the saccharification efficiency to 55% in 96 h. The reducing sugar yield obtained in the present study was compared with previous reports (Table 3). In contrast, the commercial enzyme from *Aspergillus niger* (0.8 U mg^−1^) showed 8.51 to 13.07% efficiency at 72 h, which declined thereafter (Fig. 10).

**Table 3:** Comparison of different pretreatment methods and saccharification efficiency using thermophilic cellulases in CCB

Parameters	Bu et al. [52]	Sukai and Kana [53]	Thangavelu et al. [10]	This study
Biomass	Corn cob	Corn cob	Corn cob	Corncob
Source of cellulase	Cellic CTec2	Celluclast	–	CMCPS1
pH	–	–	–	3–9
Temperature °C	–	–	–	30–90
Metal/solvent tolerance	–	–	–	Zn^2+^; acetone, methanol and isopropanol
Delignification (%)	17.82	63.61	47.44	54.1
Enzyme loading	10 FP units g^−1^	12 FP units g^−1^ and 15 CBH IU g^−1^	–	40 IU g^−1^; 20 IU g^−1^
Solid loading	10%	2.5%	–	5%
Saccharification conditions	47 °C pH 4.8	50 °C	–	50 °C pH 5.0
Reducing sugar yield	–	1.10 g g^−1^	–	1.37 g g^−1^ (40 IU g^−1^) 0.99 g g^−1^ (20 IU g^−1^)
Efficiency	Tenfold increase	–	–	55%

## Discussion

Thermostable cellulases offer potential merits in LCB valorization by increasing its solubility and resulting in higher reaction velocities and a paramount reduction in enzyme load [13, 14]. In addition, thermostable cellulases offer other advantages of shorter duration of hydrolysis, decreased contamination risks, increased productivity, and a more predominantly reduced cost of energy for cooling after the pretreatment process. With a prospect to search for novel thermostable GHs, it is now widely accepted that thermophilic microbes growing at a temperature of 50 to 80 °C are the nature’s reserve for hyperactive thermo-, alkali- and solvent-tolerant cellulases [15–19]. The present study isolated thermotolerant cellulases producing thermophilic bacterial strain *B. aerius* CMCPS1 obtained through the biotrap-based enrichment of paddy straw in the hot springs (Manikaran) of India. Previously, a variety of microorganisms belonging to the genera *Bacillus, Geobacillus, Thermotoga, Caldibacillus, Acidothermus, Caldocellum,* and *Clostridium* were reported to produce thermostable cellulases [2]. Furthermore, an enhanced endoglucanase production by *Bacillus aerius* S52 on mixed lignocellulosic substrates was attempted [20]. However, there exist no previous reports of thermophilic *B. aerius* producing the thermotolerant cellulase, as the majority were reported in *B. licheniformis* and *B. subtilis* [2, 21].

It is crucial to determine the GHs of thermophilic bacterium *B. aerius* CMCPS1 on substrates, such as filter paper, CMC, Avicel, and cellobiose. Cellulases constitute a multi-enzyme complex encompassing at least three components, namely exoglucanases, endoglucanases, and β-glucosidases, for efficient conversion of cellulose to glucose. In the present study, *B. aerius* CMCPS1 produced all three cellulases: endoglucanase (2.0 IU mL^−1^), exoglucanase (1.5 IU mL^−1^), and β-glucosidase (1.0 IU mL^−1^) at 48 h under submerged fermentation. Before this, a variety of thermophilic bacteria were identified as GH producers, including *B. circulans* [22], *B. subtilis* [23], *B. licheniformis,* and *B. tequilensis* [21]. Higher levels of aryl II β-glucosidase activity was found on cellulose than aryl III β-glucosidase activity. Conversely, on cellobiose, aryl III β-glucosidase activity increased, and it did not hydrolyze CMC. Because these enzymes are located externally in the *Bacillus* sp., they prefer to grow more on the cellulosic substrates than cellobiose [24]. Similarly, endoglucanase was more external while localizing the cellulase components in *Pseudomonas* sp. The growth preference of cell-bound aryl II β-glucosidases of CMCPS1 has to be elucidated. It has been noticed that the prolonged incubation of CMCPS1 after 48 h declined the enzyme activity and biomass conversion, which could be attributed to the consequence of random lethal events, including cellular fragmentation in the death phase and release of intracellular proteases into the fermentation broth [25].

Endoglucanases with carbohydrate-binding modules (CBMs) depolymerize crystalline cellulose. In the present study, *B. aerius* CMCPS1 exhibited a significant *celB* amplicon (650 bp), suggesting the presence of an inherent gene for endo β 1-4 glucanase, belonging to the CBM family 46. The presence of *celB* from *B. halodurans* of CBM 46 has been reported earlier [26]. However, *celS* was not found in *B. aerius* CMCPS1. Our previous finding [21] confirmed the presence of cellulose-binding operons of size 250 bp (*celS*) domain in thermophilic bacterial strains of *B. tequilensis* isolated from the hot springs of Himachal Pradesh. In addition, the β-glucosidase-specific primers generated a significant amplicon size of 1500 bp, which further confirmed the presence of multidomain GH-encoding operons. The majority of the hyperthermophilic microbes did not degrade crystalline cellulose due to the absence of a CBM [27]. However, the presence of a multidomain thermophilic cellulase of CMCPS1 favors the degradation of LCB [28]. Hence, it can be concluded that *B. aerius* CMCPS1 possessing multidomain GH cellulase allows efficient cellulose depolymerization at high temperatures as a single source of the biocatalyst.

Partial purification of cell-free culture supernatant of CMCPS1 revealed multiple banding patterns, which could be attributed to endoglucanases of cytoplasm, endoglucanases from periplasm, or aryl-β glucosidase II [21]. In the present study, the GHs were extracellular, as evidenced by the agarose cell diffusion assay. Hence, these endoglucanases could be bound to cells and may be exoenzymes, as observed in our previous report [21] and by Li et al. [29].

The properties of cellulases, particularly enzyme deactivation and stability under adverse temperature and pH conditions, are essential for industrial applications. The broader pH stability of CMCPS1 confirms its tolerance to alkali. Alkaline cellulases from alkaliphilic *Bacillus* strain KSM-635, which is active at pH values higher than 8.0 under mesophilic conditions (30–37 °C), has already been reported [30]. Moreover, thermostable alkaline cellulases (pH 3.0–9.0) from *Bacillus licheniformis* 380, isolated from compost, have been purified and characterized [31]. In addition, acid-tolerant cellulases (pH 4.8) have been reported by several other studies on *Nectria catalinensis* [32]. Hence, the results suggest the possibility of using alkali-tolerant cellulases isolated from *B. aerius* CMCPS1 for potential application in the detergent industry.

Thermal inactivation of GHs is often encountered in most biorefineries. The present study envisaged the thermal stability of purified cellulase over a temperature ranging from 30 to 90 °C. At 80 °C and 90 °C, the CMCase activity declined rapidly, possibly because of protein instability to higher temperatures, leading to thermal denaturation. Cellulases with an activity up to 40 °C cannot saccharify efficiently because the enzymatic hydrolysis at ≤ 50 °C is often incomplete. Hence, higher thermostable cellulases are preferred. It has been reported that cellulases showed broader thermal stability at different incubation times, and their activity also retained 90% and 60% of the maximum activity at 60 °C and 80 °C, respectively [33]. Moreover, the CMCase of *B. aerius* CMCPS1 retained > 50% of activity beyond 80 °C. Several other thermophilic strains have been shown to produce thermostable cellulases; however, the activity was not retained at increased temperatures for more prolonged incubation [34].

In the present study, a strong inhibitory effect of cellulase activity in the presence of Fe^2+^ and Cu^2+^ ions and a more stable activity owing to acetone was observed following the earlier reports in *Bacillus amyloliquefaciens* DL-3 [35] and *Bacillus vallismortis* RG-07 [36]. The inhibition of cellulase activity by the divalent metal cations Cu^2+^ and Fe^2+^ ions might be due to (i) the prevalence of their competition with protein-associated cations, resulting in reduced metalloenzyme activity; (ii) binding with thiol groups and interaction with carboxyl or imidazole groups of amino acids [37]. However, the thermostable cellulase of *B. aerius* CMCPS1 is moderately tolerant to Zn^2+^ and Ca^2+^. In contrast, *Paenibacillus* sp. strain B39 showed the maximum enzyme activity in the presence of Ca^2+^, Mg^*2*+^, and Na^+^ ions [38].

Enzyme-catalyzed reactions can be significantly enhanced in organic solvents than in natural aqueous water-based media, which offer numerous potential applications in several industrial processes. The cellulase of *B. aerius* CMCPS1, in the presence of acetone, exhibited much higher relative activity, whereas polar solvents showed a marginal reduction. A previous report demonstrated that except for benzene, solvents propanol, ethanol, acetone, methanol, and cyclohexane increased the cellulase activity of *B. vallismortis* RG-07 [36]. Similarly, an organic solvent-stable cellulase of *B. halodurans* CAS1 with enhanced activity in the presence of organic solvents (25% v/v) has also been reported [39]. The stimulation of enzyme activity by organic solvents may be due to the formation of intermediary compounds of residues of non-polar hydrophobic solvents. The cellulase is thereby maintained in an open conformation, resulting in stimulated activation. We, therefore, suggest that the cellulase of thermophilic *B. aerius* CMCPS1 is highly stable in the presence of both hydrophilic and hydrophobic organic solvents at 30% v/v.

In the present study, the enzymatic pretreatment of CCB in an HCR-LccH resulted in a significant reduction in lignin (54.1%) and hemicellulose (6.57%), and increase in cellulose (24.26%), which confirms our previous finding [10]. With the pre-treated CCB, at 5% solid loading rate, we attained a saccharification efficiency of 54.59% using partially purified cellulase (40 U mL^−1^) of CMCPS1 at 96 h. Cellulase, a complex of three different enzymes, acts in synergism and a cooperative association, producing substrates for each other [17, 40]. The decrease in the sugar yield at low enzyme loading beyond the maximum solid loading concentration might be attributed to the presence of inhibitors, as observed earlier [41]. The accumulated cellodextrin and cellobiose with a lower degree of polymerization might inhibit hydrolysis. A saccharification yield of 46% was also obtained previously using cellulase from *Trichoderma citrinoviride* using perennial grass *Artemisia annua* under pretreated conditions [42]. Similarly, the one-pot consolidated bioprocessing of ionic liquid-pretreated pine needles by *B. subtilis* (SV1) achieved a maximum saccharification efficiency of 65.9% [43]. The difference in sugar yield might be caused by the pretreatment method or nature of biomass used. In the present study, HCR-LccH pretreatment resulted in a higher degree of delignification. The cellulase of CMCPS1 has more accessibility to cellulose, at 5% solid loading rate, and an enzyme loading rate of FP units 40 U g^−1^ recovered higher sugar from CCB compared to the previous reports [10, 52, 53]. Yet this is the first report on thermo-alkali-tolerant multi-domain GH of *B. aerius* CMCPS1 and its saccharification efficiency in HCR-LccH-pretreated biomass.

## Conclusions

Thermophilic cellulolytic bacterial strain CMCPS1, isolated from an enriched biomass sample at the hot springs of Himachal Pradesh, was identified as *Bacillus aerius* CMCPS1. In general, the stability of the partially purified cellulase of CMCPS1 over a range of pH, temperature, metal ions, and organic solvents offers a wide scope for its applications in biorefineries to produce sugars and concomitant fermentation products. The activity over a wide range of pH (3–9) and temperature (30–90 °C) clearly indicates the thermoalkaline nature of cellulase from CMCPS1. The saccharification efficiency of cellulase from CMCPS1 was 55% on HCR-LccH-pretreated corncob at the maximum enzyme and substrate loading rate of 40 U g^−1^ and 5%, respectively. There exists further scope of enhancing the saccharification efficiency by staggered enzyme loading, making an enzyme cocktail, and optimizing the conditions. Moreover, the strain is unique with respect to cellulase activation in the presence of several hydrophobic solvents, which make the strain a cost-efficient resource for thermo-alkali and solvent-tolerant cellulase production and subsequent bioconversion of LCB into fuels and platform chemicals.

## Methods

### In situ enrichment, isolation, screening, and identification of thermophilic cellulase- producing bacteria

An in situ enrichment with several cellulose-rich natural substrates was performed at the mouth of hot springs in Manikaran (~ 95 °C), Himachal Pradesh, India (32.0268° N, 77.3511° E). Perforated tubes with the conical bottom (15 mL) containing 200 to 300 mg of substrates (paddy straw, banana fiber, banana pseudostem, rice husk, palm fronds) placed in the spring for 2 weeks were used for isolating the microbes. Isolation, screening, and molecular characterization of thermophilic bacteria were performed as described previously [21]. Thermophilic cellulase-producing bacteria were isolated by dilution plate on a basal medium supplemented with 1% CMC. The plates incubated at 50 °C for 24 to 48 h were flooded with 0.1% Congo red, followed by destaining with 1 M NaCl [44]. The hydrolytic capacity of the bacterial isolates was calculated as given below:

$${\text{Hydrolytic}}\;{\text{capacity }} = {\text{ diameter}}\;{\text{of}}\;{\text{the}}\;{\text{clearing}}\;{\text{zone }}\left( {\text{cm}} \right)/{\text{diameter of the colony }}\left( {\text{cm}} \right).$$

The cellulolytic activity was compared with that of standard cultures *B. tequilensis* VCB1 (NAIMCC–B-02117) and *B. licheniformis* KBFB3 (NAIMCC–B-02118), obtained from the National Bureau of Agriculturally Important Microbes (NBAIM, ICAR-India).

### Cellulase production by *B. aerius* CMCPS1 under submerged fermentation

#### Growth and cellulase production by CMCPS1

A single colony inoculated into 5 mL of Luria–Bertani (LB) broth was incubated overnight at 50 °C and further transferred into 100 mL of basal media amended with 1% CMC as the substrate [45]. The growth was monitored periodically for 78 h at 50 °C, and the OD_600_ was measured at every 4 h interval. The growth rate was determined using the commercially available software GraphPad Prism 5.0.

#### Preparation of crude enzyme extract

The overnight-grown culture (OD_600_ of 0.6) was inoculated into 50 mL of BPS-X production medium as described previously [21] supplemented with 1% CMC for cellulase production [46]. The flasks were incubated at 50 °C in an orbital shaker at 110 rpm for 168 h. The supernatant collected at every 24-h interval was centrifuged at 10,000 rpm for 10 min at 4 °C, and the cell-free culture supernatant served as the source enzyme for the following assays.

#### Partial purification of cell-free crude cellulase

The bacterial culture was harvested by centrifugation at 10,000 rpm for 20 min at 4 °C. The supernatant saturated with 80% ammonium sulfate was frozen and incubated for 12 h. The incubated sample was centrifuged at 10,000 rpm for 20 min at 4 °C to remove salts. The pellet resuspended in 100 mM phosphate buffer, pH 7.0, was assayed for enzyme activity and determined for protein content.

#### Enzyme activity measurement

Assays for total cellulases (FPA), CMCase, Avicelase, and cellobiase from cell-free crude extract were performed according to standard protocols. Respective substrates, i.e., filter paper, CMC, Avicel, and cellobiose, were used to monitor the enzyme activity as described [21]. One unit of enzymatic activity is defined as the amount of enzyme that releases 1 µmol of reducing sugars (measured as glucose) per mL per min.

### Screening for *cellulase* genes

Thermophilic CMCPSI strain was further screened by PCR for cellulase-encoding genes using gene-specific primers (Additional file 1: Table S1), and the amplicons were resolved in 1% agarose gel.

### CMCase activity on an agarose plate

CMC (0.1%) containing agarose plates were prepared with wells. Approximately 20 µL of the partially purified enzyme was added to the wells drilled on CMC agarose plates and incubated overnight at 50 °C for 12 h. The dishes were washed with distilled water, and staining (0.1% Congo red solution) and destaining (1 M NaCl) were performed as mentioned above to detect the clearing zones around the wells due to the hydrolytic activity [47].

### Sodium dodecyl sulfate–polyacrylamide gel electrophoresis analyses

Sodium dodecyl sulfate–polyacrylamide gel electrophoresis (SDS-PAGE) was performed with 12% resolving and 4% stacking gels, according to Laemmli’s method [48]. After electrophoresis, protein bands were detected as described previously [21].

### Native PAGE and zymogram

The partially purified culture supernatants were run on 12% non-denaturing PAGE. The gel was placed in the agarose plate containing 0.1% CMC and incubated for 1 h at 50 °C [47].

### Protein determination

The protein concentration was determined [49] by mixing 1 mL of Bradford’s reagent (BioRad) with 50 µL of the sample and read in a multimode microtiter plate reader (SpectraMax@i3x) at 595 nm using bovine serum albumin as the standard. The specific activity of CMCase was calculated and expressed in terms of IU per mg of protein.

### Optimization of assay conditions

#### Effect of temperature, pH, solvent, and metals on enzyme activity

The optimum temperature, pH, solvent, metal ion concentration for the enzyme activity were monitored. The thermal stability was monitored by incubating the enzymes with 1% CMC in 100 mM phosphate buffer under different temperatures ranging from 30 to 95 °C. The optimum pH of the purified cellulase was determined by pre-incubating the mixture of the purified enzyme and 1% (w/v) CMC in the presence of appropriate buffers; 100 mM acetate buffer (pH 3.0), 100 mM phosphate buffer (pH 7.0), and 100 mM Tris–HCl buffer (pH 8.5), and ammonia buffer (pH 9.5). The reaction mixtures were pre-incubated at 50 °C for 60 min and assayed for the activity. The pH stability was determined by incubating the purified enzyme in respective buffers having different pH ranges from 3.0 to 10.0 at 50 °C for 5 h. The residual activity of each sample for hydrolysis of CMC was subsequently estimated under assay conditions, as described above.

The effect of various metal ions (5 mM) on the enzyme activity was determined using NH_4_Cl, KCl, CaCl_2_, ZnSO_4_, CuSO_4_, and FeSO_4_. The enzyme was incubated with different metals at 50 °C for 1 h, and the cellulase activity was determined under standard assay conditions.

For the solvent tolerance test, the partially purified enzyme was incubated with 30% (v/v) of different organic solvents (methanol, ethanol, acetone, isopropanol, *n*-hexane, and toluene) in screw-capped tubes and incubated at 50 °C for 1 h. The residual cellulase activity (%) was determined under standard assay conditions, as described above.

### Pretreatment of corncob biomass by HCR-LccH

The CCB was dried to remove the moisture, and the size was reduced in a sequence shredder, pin mill, and grinder. The powdered biomass was sieved using ASTM 70 sieve as per the ASTM E11-13 procedure. The size of the biomass particles used for the pretreatment was less than 212 µm. Pretreatment of CCB was performed in an HCR at a 5% solid loading rate with laccase from *H. hirta* LccH [12] at 6.5 U g^−1^ for 60 min [10]. The compositional analyses of the feedstock CCB before and after pretreatment was determined as per the standard protocols of NREL, 2004 [50].

### FT-IR analysis

The FT-IR spectra of HCR-LccH-pretreated CCB were obtained on an FT-IR instrument (FT-IR 6800 JASCO, Japan) using KBr discs containing 1% finely ground samples. The absorbance spectra were recorded with a spectral resolution of 4 cm^−1^ and 64 scans per sample between wavenumbers 4000 and 400 cm^−1^.

### Enzymatic saccharification of HCR-LccH-pretreated CCB

Saccharification was performed by suspending the pretreated biomass in 0.1 M sodium citrate buffer (pH 5.0) in capped polycarbonate flasks, and the substrate concentration was maintained at 10% (w/v). The crude CMCase was added in different doses (20, 30, and 40 U g^−1^). Hydrolysis was performed at 50 °C for 120 h at 120 rpm in a water bath shaker and slightly modified from the method described previously [51]. Samples drawn at every 24 h were centrifuged, and the supernatants were analyzed for reducing sugars at 540 nm [39]. Enzymatically pre-treated CCB with commercial cellulase of *A. niger* (Sigma cat no. C1184) served as the control.

## Supplementary information

**Additional file 1: Table S1.** Primers used for screening GHs.

## Data Availability

All data of this manuscript are included in the manuscript. No separate external data source is required. Any information required will be provided by communicating with the corresponding author via the official mail: usiva@tnau.ac.in.

## Abbreviations

CMCPS1: Bacterial isolate obtained by in situ enrichment of lignocellulose substrates from hot springs of Himachal Pradesh
LccH: Crude laccase from *Hexagonia hirta* MSF2
FPA: Filter paper assay
HCR: Hydrodynamic cavity reactor
LCB: Lignocellulosic biomass
SDS-PAGE: Sodium dodecyl sulfate–polyacrylamide gel electrophoresis
GHs: Glycosyl hydrolases
CBM: Carbohydrate-binding module
