# Supplementary material for: Thermotolerant glycosyl hydrolases-producing Bacillus aerius CMCPS1 and its saccharification efficiency on HCR-laccase (LccH)-pretreated corncob biomass
Source: Biotechnol Biofuels. 2020 Jul 14;13:124. doi: 10.1186/s13068-020-01764-2 (PMC7362481; doi:10.1186/s13068-020-01764-2)
Supplement: Supplementary file 1 — Additional file 1: Table S1. Primers used for screening GHs. [file 13068_2020_1764_MOESM1_ESM.doc]

**Additional File**

| **rimer name** | **Primer sequences** | **References** |
| --- | --- | --- |
| CelS F  CelS R | 5’-GCCCTTGGTGTCCTTGATC 3’  5’- CTCTACAACAACATCTGGGG 3’ | Bischoff ***et al.***, 2006  Saarilhati***et al.****,* 1990 |
| CelB F  CelB R | 5’-GTCCAGAACAACGCTGGGG -3’  5’-CGTTCTGCCACGGCTCGA-3’ |
| BGL_H F  BGL_H R | ATGACCGAACAGACCAAAAAA  CTACAGGCTTTCGCCGTT | Saarilhati***et al.****,* 1990 |

**Table S1: Primers used for screening GHs**
